# Retraction: Rpb3 promotes hepatocellular carcinoma through its N-terminus

**DOI:** 10.18632/oncotarget.28784

**Published:** 2025-12-31

**Authors:** Zhe-Ping Fang, Bei-Ge Jiang, Fa-Biao Zhang, Ai-Dong Wang, Yi-Ming Ji, Yong-Fu Xu, Ji-Cheng Li, Wei-Ping Zhou, Wei-Jie Zhou, Hai-Xiong Han

**Affiliations:** ^1^Department of Hepatobiliary Surgery, Taizhou Hospital of Zhejiang Province, Wenzhou Medical University, Linhai 317000, China; ^2^Eastern Hepatobiliary Surgery Hospital, Second Military Medical University, Shanghai 200438, China; ^3^Institute of Cell Biology, Zhejiang University, 866 Yu-Hang-Tang Road, Hangzhou 310058, China; ^4^Department of Biologic and Materials Sciences, University of Michigan School of Dentistry, Ann Arbor, MI 48109, USA; ^*^Contributed equally to this work

**This article has been retracted:** Oncotarget has concluded its investigation of this paper. Oncotarget was contacted by the corresponding author, who stated, ‘Upon recent review of our published work, we have come to the realization that during the preparation of the figures for this article, an unfortunate error occurred. We inadvertently used images that belong to another research team. This oversight on our part has raised concerns about the reliability of our findings. Given that a considerable amount of time has passed since the original research was conducted, we find ourselves in a position where we are unable to re-validate the study and ensure the accuracy of our conclusions. Therefore, after careful consideration and in the interest of maintaining the integrity of the scientific record, we respectfully request the retraction of our article.’ Oncotarget has since determined that all 4 images in question appear in Figure 4A, and were duplicated in Figure 6A of another paper published later [1], using different labels. In light of these circumstances, the Editorial Board has made the decision to retract this article. All authors have agreed with this decision.

Original article: Oncotarget. 2014; 5:9256–9268. 9256-9268. https://doi.org/10.18632/oncotarget.2389
